# Draft Genome Sequence of *Sporosarcina globispora* W 25^T^ (DSM 4), a Psychrophilic Bacterium Isolated from Soil and River Water

**DOI:** 10.1128/genomeA.01230-15

**Published:** 2015-10-22

**Authors:** Jie-ping Wang, Bo Liu, Guo-hong Liu, Rong-feng Xiao, Xue-fang Zheng, Huai Shi, Ci-bin Ge

**Affiliations:** Agricultural Bio-Resources Research Institute, Fujian Academy of Agricultural Sciences, Fuzhou, Fujian, China

## Abstract

*Sporosarcina globispora* W 25^T^ (DSM 4) is a Gram-positive, round-spore-forming, and psychrophilic bacterium. Here, we report the 5.66-Mb genome sequence of *S. globispora* W 25^T^, which will accelerate the application of this psychrophile and provide useful information for genomic taxonomy and phylogenomics of *Bacillus*-like bacteria.

## GENOME ANNOUNCEMENT

As early as 1967, the psychrophilic strain W 25^T^ was isolated and initially classified as *Bacillus globisporus* by Larkin and Stokes (1). In 2001, Yoon et al., reclassified *B. globisporus* as *Sporosarcina globispora* comb. nov. (2). The strains of *S. globispora* can grow and sporulate at 0°C with a maximum temperature of 20 to 25°C for growth (1, 2). Therefore, *S. globispora* strains can produce psychrophilic enzymes, such as adenylate kinases that maintain cellular homeostasis of A, ADP, and ATP (3, 4). Some *S. globispora* strains such as N75 and C11 can synthesize cyclic tetrasaccharide or pentasaccharide from starch or alpha-1,4-glucan catalyzed jointly by 6-alpha-glucosyltransferase and 3-alpha-isomaltosyltransferase (5–7). More recently, the *S. globisporus* strain Q12 was found to possess silicate mineral-solubilizing and potassium-bearing mineral-solubilizing activities, thereby making it a candidate for the production of microbial fertilizers (8, 9).

Given the physiological properties and application prospects, and because there is no available genomic information on *S. globispora*, its type strain W 25^T^ was selected as one of the research objects in our “genome sequencing project for genomic taxonomy and phylogenomics of *Bacillus*-like bacteria.” Here, we presented the high-quality draft genome sequence of *S. globispora* W 25^T^ (DSM 4).

The genome sequencing of *S. globispora* W 25^T^ (DSM 4) was performed via an Illumina Hieseq 2500 system. Two DNA libraries with insert sizes of 500 and 5,000 bp were constructed and sequenced. After filtering of the 1.44-Gb raw data, the 1.39-Gb clean data was obtained, providing approximately 200-fold coverage. The reads were assembled via SOAPdenovo version 1.05 (10), using a key parameter K setting at 71. Through the data assembly, 21 scaffolds with total length 5,673,948 bp were obtained, and the scaffold *N*_50_ was 5,539,694 bp. The average length of the scaffolds was 270,188 bp, and the longest and shortest scaffolds were 5,539,694 bp and 566 bp, respectively. Total 89.31% clean reads could be aligned back to the genome, which covered 99.83% f the sequence.

The annotation of the genome was performed using the NCBI Prokaryotic Genomes Automatic Annotation Pipeline (PGAAP) (http://www.ncbi.nlm.nih.gov/genome/annotation_prok/) utilizing GeneMark, Glimmer, and tRNAscan-SE tools (11). A total of 5,580 genes were predicted, including 5,147 coding sequences (CDS), 287 pseudo genes, 116 tRNAs, and 29 rRNA genes. There were 3,687 and 2,847 genes assigned to the COG and KEGG databases, respectively. The average DNA G+C content was 40.46%, agreeing with the values 39.7 mol% (Bd) and 39.8 mol% (*T*_m_) acquired previously (1). 

### Nucleotide sequence accession numbers.

This whole-genome shotgun project has been deposited at DDBJ/EMBL/GenBank under the accession no. LGUF00000000. The version described in this paper is version LGUF01000000.
